# Clinical significances of preoperative serum interleukin-6 and C-reactive protein level in operable gastric cancer

**DOI:** 10.1186/1471-2407-9-155

**Published:** 2009-05-20

**Authors:** Do-Kyong Kim, Sung Yong Oh, Hyuk-Chan Kwon, Suee Lee, Kyung A Kwon, Byung Geun Kim, Seong-Geun Kim, Sung-Hyun Kim, Jin Seok Jang, Min Chan Kim, Kyeong Hee Kim, Jin-Yeong Han, Hyo-Jin Kim

**Affiliations:** 1 Departments of Internal Medicine, Dong-A university College of Medicine, Busan, Korea; 2 Departments of Surgery, Dong-A university College of Medicine, Busan, Korea; 3 Departments of Laboratory Medicine, Dong-A university College of Medicine, Busan, Korea

## Abstract

**Background:**

The interleukin-6 (IL-6) pathway is one of the mechanisms that link inflammation and angiogenesis to malignancy. Because the C-reactive protein (CRP) is a representative marker for inflammation, CRP has recently been associated with the progression of disease in many cancer types. The principal objective of this study was to determine the preoperative serum levels of IL-6 and CRP in gastric carcinoma, and to correlate them with disease status and prognosis.

**Methods:**

A total of 115 patients who underwent gastrectomy were enrolled in this study. Serum levels of IL-6 were assessed via Enzyme-Linked Immuno-Sorbent Assay (ELISA), and CRP was measured via immunoturbidimetry. Histological findings included tumor size, depth of tumor invasion, lymph node (LN) metastasis, and TNM stage (6th AJCC Stage Groupings: The staging systems; Primary tumor, regional LN, metastasis).

**Results:**

Increases in cancer invasion and staging are generally associated with increases in preoperative serum IL-6 levels. IL-6 and CRP levels were correlated with invasion depth (*P *< 0.001, *P *= 0.001), LN metastasis (*P *< 0.001, *P *= 0.024) and TNM stage (*P *< 0.001, *P *< 0.001). The presence of peritoneal seeding metastasis is associated with IL-6 levels (*P *= 0.012). When we established the cutoff value for IL-6 level (6.77 pg/dL) by ROC curve, we noted significant differences in time to progression (TTP; *P *< 0.001) and overall survival (OS; *P *= 0.010). However, CRP evidenced no significance with regard to patients' TTP and OS levels. Serum IL-6 levels were correlated positively with CRP levels (*r*^2 ^= 0.049, *P *= 0.018).

**Conclusion:**

Preoperative serum IL-6 and CRP levels might be markers of tumor invasion, LN metastasis, and TNM stage. Preoperative high IL-6 levels were proposed as a poor prognostic factor for disease recurrence and overall survival in patients with gastric cancers.

## Background

Interleukin-6 (IL-6) is a multi-poietic cytokine that induces the growth and differentiation of immune cells, the production and expression of other cytokines, and acute-phase protein synthesis. IL-6 also exerts several effects on cancer cells [1,2].

In cancer, IL-6 is generally known to be involved in host defense mechanisms. IL-6 binds to the IL-6 receptor, activates the Janus kinase (JAK), and subsequently phosphorylates the signal transducers and activators of transcription (STAT). The phosphorylated STAT gene translocates into the nucleus and activates the target gene (JAK/STAT) pathway. Suppressor of cytokine signaling-1 (SOCS-1) is one of the STAT-activated genes, which is upregulated by IL-6 and is involved in the downregulation of the JAK/STAT pathway [3-5]. In many cancer types, recent studies have demonstrated that the hypermethylation of SOCS-1 is not controlled by the JAK/STAT pathway, and IL-6 cannot perform a role in cancer defense; on the contrary, it is involved in cancer development [3,6].

In the development and progression of cancer, angiogenesis is a crucial and essential process. IL-6 is associated with angiogenesis by virtue of its ability to induce the mRNA of vascular endothelial growth factor (VEGF), which is typically a direct angiogen [1]. Additionally, IL-6 activates the Rho protein, which is associated with cell-cell adhesion and invasion in malignancy [7].

C-reactive protein (CRP) is a representative marker for inflammatory conditions, and performs a crucial anti-infection function in the immune system. In many cancers, it has been reported that chronic inflammation is involved with malignant change, and the risks of cancer are increased when pre-diagnostic CRP levels are high [8]. Cancer invasion begins with inflammation around cancer cells. Thus, it has been reported that serum CRP levels are higher in cases of invasive cancer than in cases of non-invasive cancer [9,10].

Gastric cancer is the fourth most-frequent source of morbidity worldwide, and the second most-frequent source of mortality [11,12]. In Korea, gastric cancer is the most frequent source of morbidity among malignant tumors [13]. These high levels of gastric cancer mortality can be attributed to the high incidence of serosal invasion, direct invasion into the adjacent organs, peritoneal seeding, lymph node metastasis, and distant metastasis of gastric cancer. In cases of gastric cancer, the failure of downregulation of the JAK/STAT pathway due to the hyper-methylation of SOCS-1 has been surveyed, and angiogenesis has been identified as an indispensable process for high tendencies toward malignancy [3,14,15]. The principal objective of this study was to determine the relationship between serum IL-6 and CRP levels and malignant tendencies and prognosis in gastric cancer patients.

## Methods

### Patients

From March 2005 to August 2008, a total of 115 patients with histologically-confirmed gastric carcinomas from surgically resected specimens of gastric tumors at the Dong-A University Hospital were enrolled in this study. After operation, the disease status of patients was evaluated via gastroendoscopy and abdominal computed tomography (CT). During the first 2 years, we checked CT and endoscopy at six-month intervals, and afterwards CT and endoscopy were conducted every 12 months. We also conducted abdomen CT when we observed abdominal distension, acute abdominal pain, severe diarrhea, and chest X-ray abnormalities suggestive of lung metastasis. Endoscopy was conducted in cases in which patients complained of unexplained abdominal discomfort and dyspepsia. On average, in the stage III and IV patients, the first evaluation was conducted within 3 months after operation. All patients provided informed consent, and the hospital review board approved the study.

### Assay of serum IL-6 and CRP

Venous blood sampling was conducted within 7 days before the patients underwent operations. The blood collected for IL-6 serum level assessments was collected in plain tubes, and the levels of serum IL-6 were measured using commercially available enzyme-linked immunosorbent assays (ELISAs) (Quantikine h IL-6 Immunoassay, R&D Systems, USA). The blood samples were centrifuged for 10 min at 3,000 r/min at -4°C. The serum was subsequently removed and stored at -80°C until biochemical analysis. The blood samples for CRP analysis were collected in serum separation tubes, and the serum CRP levels were measured via immunoturbidimetry (Denka Seiken Co. Ltd., Japan).

### Statistical analysis

Serum levels of IL-6 and CRP were expressed as the means ± SD. A p value of < 0.05 was considered to be statistically significant. The Spearman rho correlation coefficient (*r*) was employed to evaluate the correlation between the IL-6 and CRP levels and the clinical findings. The IL-6 and CRP cut-off values for survival analysis were determined by the ROC curve. The duration of recurrence of gastric cancer and death measured from the date of surgery was referenced against time to progression and overall survival time. Survival durations were calculated via the Kaplan-Meier method. The log-rank test was employed to compare the cumulative survival rate and time to progression in the patient groups. Linear regression analysis was conducted to estimate the relationship between the IL-6 values and CRP values. The Statistical Package for Social Sciences (SPSS) Version 15.0 for Windows was utilized to perform all statistical analyses.

## Results

### Patients' characteristics

The patients were classified by their pathologic characteristics, including tumor size, depth of tumor invasion, status of lymph node metastasis, TNM staging, and peritoneal metastasis. The patients consisted of 68 men and 47 women, with a median age of 59 years (range, 33–84 years). The characteristic data of the study population are shown in Table 1. 53 patients evidenced tumor sizes of ≥ 5 cm. The depth of tumor invasion was pT1 in 29 patients, pT2 in 48, pT3 in 32, and pT4 in 6. LN metastasis was detected in 73 patients. The postoperative stages of the patients were I, II, III, and IV in 43, 23, 30, and 19 patients, respectively. Six patients in this study had peritoneal metastasis.

**Table 1:** Patient characteristics

	No. of patients	%
Total number of patients	115	
Sex		
Male	68	59.1
Female	47	40.1
Age		
Median(Range)	59(33–84)	
Tumor size		
< 5	62	53.9
≥ 5	53	46.1
Depth of tumor invasion		
pT1	29	25.2
pT2	48	41.8
pT3	32	27.8
pT4	6	5.2
LN metastasis		
N0	42	36.5
N1	43	37.4
N2	18	15.7
N3	12	10.4
Peritoneal metastasis		
Metastasis(-)	109	94.8
Metastasis(+)	6	5.2
TNM stage		
Stage I	43	37.4
Stage II	23	20.0
Stage III	30	26.1
Stage IV	19	16.5

### Clinicopathological significance of IL-6

The relationships between IL-6, CRP levels, and clinico-pathologic variables are provided by the Spearman rho correlation coefficient (*r*) in Table 2.

**Table 2:** Correlation between the IL-6, CRP and clinicopathological parameters

	IL-6			CRP		
	
	Median ± SD	*r*	*P*	Median ± SD	*R*	*P*
					
Total	pg/ml			(mg/dl)		
**Gender**						
Male	6.83 ± 7.34	0.082	0.385	0.11 ± 0.25	-0.076	0.421
Female	7.07 ± 12.30			0.06 ± 0.43		
**Age**						
<60	6.48 ± 12.26	0.205	0.028	0.07 ± 0.30	0.152	0.104
≥ 60	7.31 ± 5.56			0.11 ± 0.38		
**Tumor size**						
<5 cm	6.48 ± 5.04	0.235	0.012	0.09 ± 0.28	0.081	0.389
≥5 cm	7.56 ± 12.93			0.09 ± 0.39		
**Tumor depth**						
pT1	6.25 ± 1.41	0.387	0.000	0.05 ± 0.10	0.311	0.001
pT2	6.60 ± 5.42			0.07 ± 0.31		
pT3	7.62 ± 16.02			0.17 ± 0.36		
pT4	9.76 ± 6.06			0.64 ± 0.64		
**LN meta**						
N0	6.36 ± 5.61	0.322	0.000	0.06 ± 0.30	0.211	0.024
N1	7.70 ± 13.99			0.11 ± 0.36		
N2	6.83 ± 2.10			0.09 ± 0.08		
N3	7.80 ± 6.96			0.19 ± 0.51		
(N1–N3)	7.56 ± 11.24	0.338	0.000	0.11 ± 0.35	0.212	0.023
**TNM stage**						
I	7.36 ± 5.52	0.425	0.000	0.06 ± 0.29	0.326	0.000
II	7.66 ± 2.24			0.07 ± 0.15		
III	11.87 ± 14.90			0.12 ± 0.30		
IV	13.92 ± 10.76			0.39 ± 0.50		
**Peritoneal**						
Metastasis(-)	6,83 ± 8.83	0.233	0.012	0.08 ± 0.29	0.175	0.061
Metastasis(+)	23.70 ± 14.55			0.41 ± 0.69		

*r*, *P*: Spearman rho correlation, IL-6: interleukin-6, CRP: C-reactive protein, LN: lymph node, TNM (6th AJCC Stage Groupings: The staging systems; Primary tumor, regional LN, metastasis)

**Figure 1:**
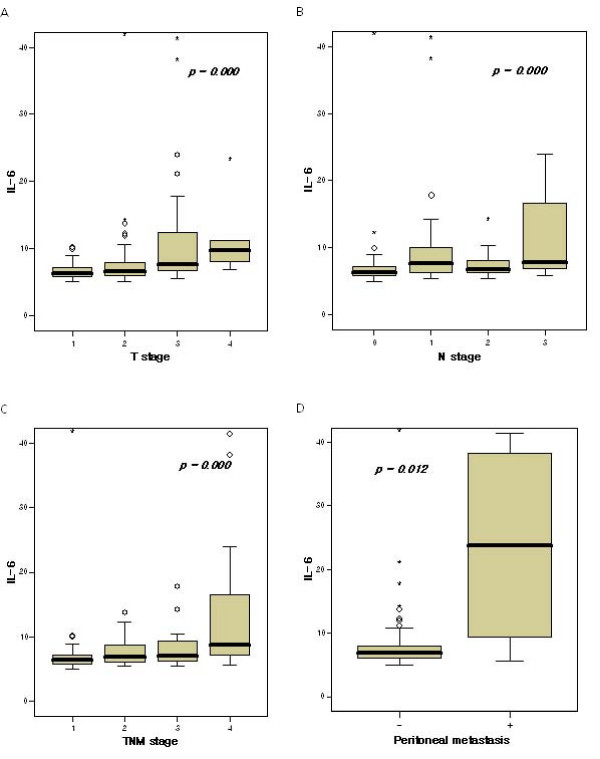
**(A) IL-6 levels according to tumor depth**. (B) IL-6 levels according to LN metastasis. (C) IL-6 levels according to the TNM stage. (D) IL-6 levels according to peritoneal metastasis. °, mild outlier (between 1.5 interquartile range (IQR) and 3.0 IQR); *, extreme outlier (more than 3.0 IQR).

We noted that IL-6 levels were significantly correlated with IL-6 levels, and tumor size with higher IL-6 levels was detected in tumors sized ≥ 5 cm (*P *= 0.0012). Additionally, with increasing degrees of tumor invasion, the median levels of IL-6 evidenced a tendency to increase, and this difference in IL-6 levels was found to be statistically significant (*P *< 0.001). In cases of LN metastasis, we also noted a significant difference between the serum level of IL-6 and LN metastasis (*P *< 0.001). The median level of IL-6 increased proportionally with the stage of the cancer (the median level of IL-6 in stage I 7.36 ± 5.52 pg/ml, stage II 7.66 ± 2.24 pg/ml, stage III 11.87 ± 14.90 pg/ml and stage IV 13.92 ± 10.76 pg/ml), and this difference was statistically significant (*P *< 0.001). Additionally, serum IL-6 levels were significantly higher in patients with peritoneal metastasis (23.70 ± 14.55 pg/ml) than in those without peritoneal seeding (6.83 ± 8.83 pg/ml, *P *= 0.012) (Figure 1).

**Figure 2:**
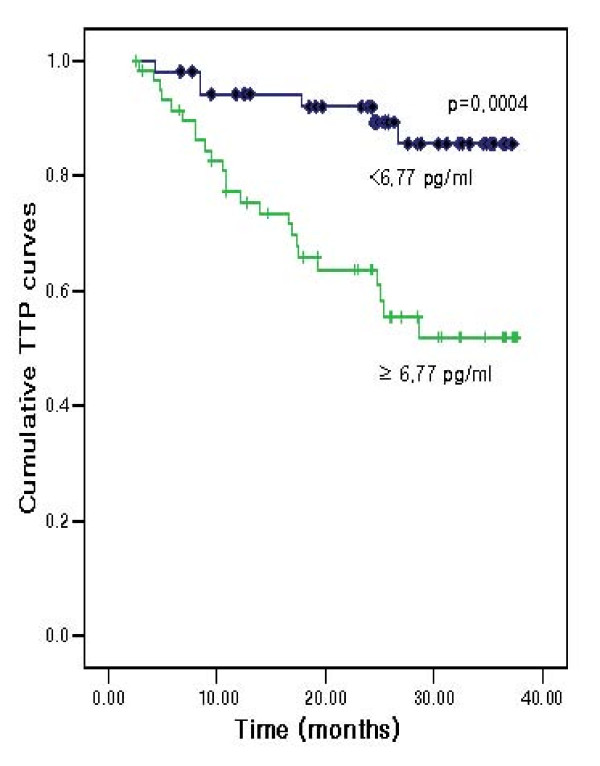
**Time to progression curve according to interleukin-6 (IL-6) level**.

**Figure 3:**
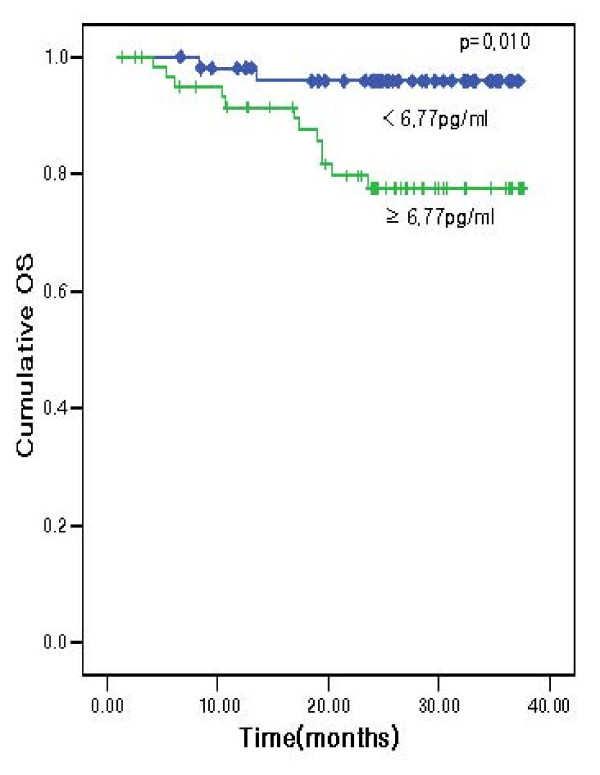
**Overall survival curve according to interleukin-6 (IL-6) level**.

The patients were divided into two groups on the basis of an IL-6 cutoff value of 6.77 pg/ml by the ROC curve with a sensitivity of 85.7% and a specificity to OS of 50.1%. The TTP values for patients with IL-6 levels in excess of 6.77 pg/ml were significantly lower than those in patients with IL-6 values of 6.77 pg/ml or less (60.0% versus 88.7%; *P *= 0.0004) (Figure 2). We also noted significant differences in the OS values (80.7% versus 96.2%; *P *= 0.010) (Figure 3). However, in multivariate analysis used to age, gender TNM stage, tumor size and peritoneal metastasis, TTP and OS showed that IL-6 was not related to TTP and OS.

### Clinicopathological significance of CRP

**Figure 4:**
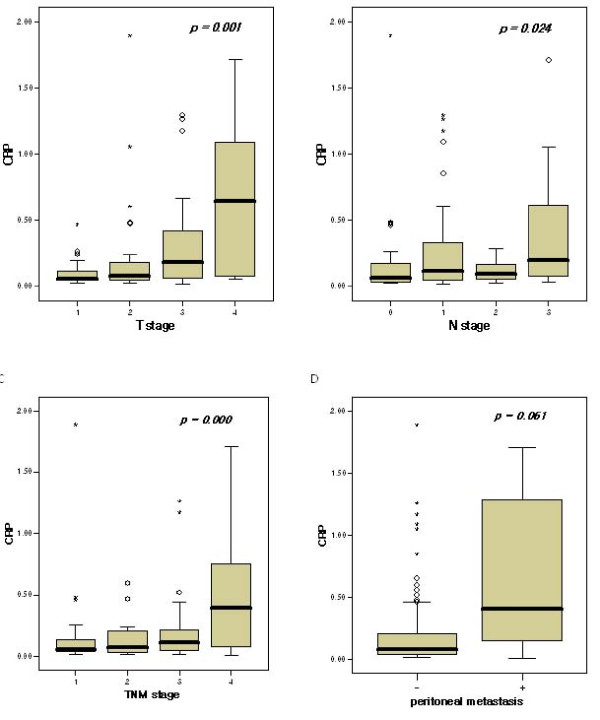
**(A) CRP levels according to tumor depth**. (B) CRP levels according to LN metastasis. (C) CRP levels according to TNM stage. (D) CRP levels according to peritoneal metastasis. °, mild outlier (between 1.5 interquartile range (IQR) and 3.0 IQR); *, extreme outlier (more than 3.0 IQR).

We noted that CRP levels did not differ significantly with tumor size (*P *= 0.039) However, levels of CRP were associated with tumor invasion depth (*P *= 0.001). We also noted significant differences in serum CRP levels between patients with lymph node metastasis and those without lymph node metastasis (*P *= 0.024). The median levels of CRP increased with increasing stage, and we also noted significant differences between the CRP level and cancer stage (the median level of CRP in stage I 0.06 ± 0.29 mg/dl, stage II 0.07 ± 0.15 mg/dl, stage III 0.12 ± 0.30 mg/dl and stage IV 0.39 ± 0.50 mg/dl; *P *< 0.001). The CRP levels did not differ significantly in patients with peritoneal metastasis (0.41 ± 0.69 mg/dl) as compared to those without peritoneal metastasis (0.08 ± 0.29 mg/dl, *P *= 0.061) (Figure 4).

0.145 mg/dl was taken as the cutoff value of CRP by ROC curve, after which the patients were divided into two groups. The sensitivity and specificity of 0.145 mg/dl as the cutoff value were 57.1% and 65.3% on OS. We noted no significant difference in the TTP values (70.7% vs 75.0%, *P *= 0.5245) and OS values (81.4% vs 91.7%, *P *= 0.0791) among the groups.

### Association between IL-6 and CRP

**Figure 5:**
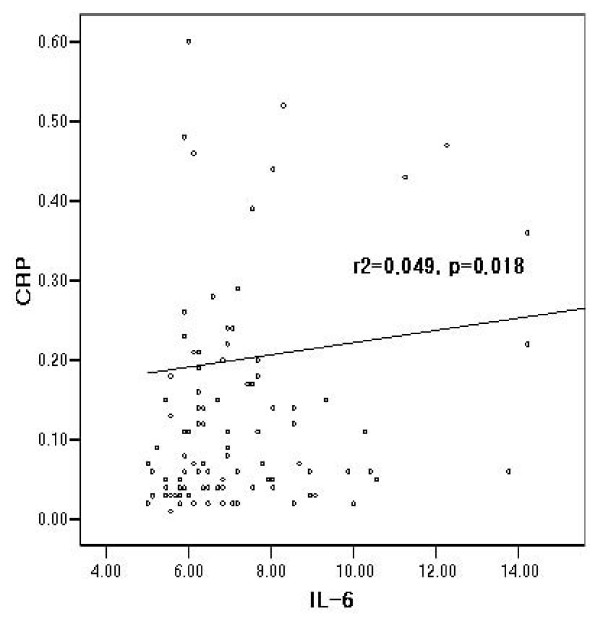
**Correlation between serum IL-6 and CRP levels in gastric cancer**.

Serum IL-6 levels were positively correlated with CRP levels (*r*^2 ^= 0.049, *P *= 0.018) (Figure 5).

## Discussion

In this study, the serum levels of both IL-6 and CRP evidenced statistically significant differences in tumor size, tumor invasion depth, and LN metastasis. In the TNM stage, as the stage of the disease increased, serum IL-6 and CRP levels were significantly higher. Additionally, the median levels of IL-6 were significantly higher in the patients with peritoneal seeding than in those without peritoneal seeding, but in CRP, this was not proven. In terms of survival rate and time to cancer progression, the results of this study showed that IL-6 levels were associated with poor prognoses. As IL-6 and CRP levels were associated strongly with disease status, in our multivariate analysis, IL-6 and CRP were not identified as independent factors. In some papers, it has been reported that IL-6 was an effective prognostic indicator in stages II and III [16]. In this study, we conducted multivariate analysis in stage II and III, but we were unable to detect any tendency that would point to IL-6 as an independent prognostic factor.

There have been a great many studies conducted concerning the values and functions of IL-6 in malignancy. The majority of these studies have shown that IL-6 levels were higher in malignancies than in non-malignancies, and increased with increasing tumor size and depth [17-19]. We detected a statistically significant relationship between IL-6 levels and existing LN metastasis. However, some studies have reported no relationship between IL-6 levels and LN metastasis [20]. LN metastasis was shown to be affected by independent predictors in cases of advanced cancers. Many previous studies have also noted that IL-6 levels increased in cases of distant metastasis, especially hepatic metastasis [17,18]. This result has been attributed to a host of mechanisms, including the autocrine and paracrine pathways. With regard to the autocrine pathway, IL-6 activated the production of IL-6 by tumor cells with the IL-6 receptor. With regard to the paracrine pathway, IL-6-stimulated stromal cells promoted the secretion of tumor growth and adhesion molecules containing VEGF and hepatocyte growth factor (HGF) [2,17,19,21].

CRP has been identified in many previous studies as a poor prognostic factor in several diseases, including coronary artery disease, chronic obstructive pulmonary disease, diabetes mellitus, myeloma bone disease, and a variety of cancers [22-30]. In many studies of cancer patients, it has been noted that elevated CRP levels are associated with tumor size, cancer stage, cancer cachexia, and poor prognosis as independent prognostic indicators [19,26,28,31]. In this paper, we described the relationship between CRP levels and tumor invasion depth, LN metastasis, and TNM stage. The operant mechanism in this regard remains unknown. However, elevations of CRP levels have been reported previously in patients with impaired T lymphocyte response, and thus this mechanism is considered to be related to the immunity system and poor survival rate [31].

CRP is generated by the liver and other organs in response to the release of IL-6 by monocytes and other immune cells [26]. Thus, when IL-6 levels increased, CRP levels also increased. Although CRP was not found, in this study, to be related to TTP and OS in cancer patients, CRP has generally been connected with IL-6, and IL-6 is associated with cancer prognosis. This study was somewhat limited in that the patients' group was restricted in patients who underwent operations. Thus, this study included more patients who could undergo surgery at a lower stage than those who evidenced contraindications for curative operations, including those patients in stage IV. In particular, as compared with another study concerning the relationship between CRP levels and cancer prognosis, a small number of advanced-stage patients were enrolled in this study [27].

Elevated serum IL-6 levels have been implicated in many different conditions characterized by chronic inflammation, including viral and bacterial infections, autoimmune disease, ischemia, diabetes mellitus, severe exercise, and malignancy [6,32]. Actually, when the IL-6 cutoff value was established at 6.77 pg/ml, the TTP and OS differed significantly between patients with IL-6 levels in excess of 6.77 pg/ml, and those with lower IL-6 levels. When the IL-6 cutoff value was established at 6.77 pg/ml, the sensitivity was 80.0% in TTP and 85.7% in OS; the specificity was 55.3% and 50.1%. Although there were many disturbance factors that complicated the elevation of IL-6 levels, the sensitivity of IL-6 in association with TTP and OS was found, in this study, to be sufficiently high. Thus, we surmise that the prognoses in gastric cancer patients with high IL-6 levels would be generally poor.

However, high IL-6 and CRP levels were related to advanced stage, including distant metastasis, in this study, and elevated IL-6 levels were associated with poor outcomes in cases of gastric cancer.

## Conclusion

Preoperative serum IL-6 and CRP levels were related to cancer stage and might be markers of tumor invasion, LN metastasis and TNM stage. Especially high IL-6 levels were prospected as a poor prognostic factor of disease recurrence and overall survival in patients with gastric cancer.

## Competing interests

The authors declare that they have no competing interests.

## Authors' contributions

D-KK performed the statistical analysis and drafted the manuscript. SYO collected the data, performed the statistical analysis with interpretation and critically revised the manuscript. H-CK, SL, KAK, BGK, S-GK, S-HK and JSJ performed the chemotherapy for patients and revised the manuscript. MCK performed the operation for patients and revised the manuscript. KHK and J-YH carried out the immunoassays. H-JK conceived of the study, and approved the final manuscript. All authors read and approved the final manuscript.

## Pre-publication history

The pre-publication history for this paper can be accessed here:

http://www.biomedcentral.com/1471-2407/9/155/prepub
